# Constipation and laxative use among people living in nursing homes in 2007 and 2013

**DOI:** 10.1186/s12877-019-1054-x

**Published:** 2019-02-08

**Authors:** Maria Gustafsson, Kristina Lämås, Ulf Isaksson, Per-Olof Sandman, Hugo Lövheim

**Affiliations:** 1 Department of Pharmacology and Clinical Neuroscience, Umeå University, SE-901 87 Umeå, Sweden; 2 Department of Nursing, Umeå University, 901 87 Umeå, Sweden; 3 Arctic Research Centre at Umeå University, 901 87 Umeå, Sweden; 4 Department of Neurobiology, Care Sciences and Society, Division of Nursing, Karolinska Institutet, 171 77 Stockholm, Sweden; 5 Department of Community Medicine and Rehabilitation, Geriatric Medicine, Umeå University, 901 87 Umeå, Sweden

**Keywords:** Constipation, Laxatives, Dementia, Nursing homes

## Abstract

**Background:**

Constipation is a common condition among older people, particularly among people living in nursing homes, and the use of drugs such as opioids is one of many factors that contribute to its high prevalence. The aim of this study was to compare the prevalence of constipation and the use of laxatives between 2007 and 2013, to analyze constipation and laxative use among people who are prescribed opioids, and to identify factors associated with constipation.

**Methods:**

In 2007 and 2013, two surveys were performed in the county of Västerbotten in Northern Sweden, comprising all those living in nursing homes. The Multi-Dimensional Dementia Assessment Scale was used to collect data regarding laxative, opioid and anticholinergic drug use, functioning in activities of daily living (ADL), cognition and symptoms of constipation. A comparison was made between 2820 people from 2007 and 1902 people from 2013.

**Results:**

The prevalence of symptoms of constipation among people living in nursing homes increased from 36% in 2007 to 40% in 2013. After controlling for age, sex, ADL, cognitive impairment and use of opioid and anticholinergic drugs, this difference was found to be statistically significant. When controlled for demographic changes, there was a statistically significant difference in the regular use of laxatives between the respective years, from 46% in 2007 to 59% in 2013. People prescribed opioids and anticholinergic drugs were at increased risk of constipation, while people with a higher ADL score were at decreased risk. Further, among people prescribed opioids and rated as constipated, 35% in 2007 and 20% in 2013 were not prescribed laxatives for regular use, a difference that was found to be statistically significant.

**Conclusions:**

The prevalence of symptoms of constipation increased between 2007 and 2013. Although there was a decrease between the years, there were still a number of people being prescribed with opioids and rated as constipated who were not treated with laxatives. This study therefore indicates that constipation remains a significant problem among people in nursing homes and also indicates that those prescribed opioids could benefit from an increased awareness of the risk of constipation and treatment, if required.

## Background

Constipation is a common condition and is particularly prevalent among the elderly with up to 40% of community-dwelling residents reporting symptoms of the condition [1, 2]. Among people living in nursing homes, prevalence up to as high as 80% has been reported [3, 4]. Chronic constipation can lead to complications such as fecal impaction, urinary retention, hemorrhoids, anal fissures and fecal incontinence [5], and it negatively impacts health-related quality of life [6]. Age-related problems such as comorbidities, dementia, decreased mobility and dehydration contribute to the increase in incidences of constipation among older adults [7]. The use of drugs is another clinical factor that impacts bowel function in the elderly [3, 7]. Medications such as urinary antispasmodics, tricyclic antidepressants and some antihistamines increase the risk of constipation because of their anticholinergic properties and the use of opioid analgesics is a risk factor for the development of constipation in the elderly in long-term care [8]. Opioid-induced constipation (OIC) is the most common adverse drug effect from opioid analgesics [9] and is estimated to affect 15–90% of patients prescribed these drugs [10]. The effects include reduced bowel tone and contractility, which prolong transit time [11].

Commonly used pharmacological drugs for the treatment of constipation include stimulant laxatives, bulking agents and osmotic laxatives. Stimulant laxatives include bisacodyl, senna, and sodium picosulfate and these exert their effects by increasing muscle contractions via enteric reflex [8]. Bulking agents such as sterculia gum and ispaghula increase the water absorption properties of the stool [12]. Osmotic laxatives include lactulose and polyethylene glycol (PEG). These drugs increase the fluid content of the bowel lumen in order to hydrate and soften the stool [8].

Studies demonstrate a high use of laxatives among older people in nursing homes and a prevalence between 55 and 67% has been found in different studies conducted from 2003 to 2008 [13–15]. One study performed among a community-dwelling elderly population showed an increased use of laxatives from 1992–1993 to 2003–2004 [16]. How the use of laxatives has changed over time in nursing homes is unclear, however, because recent data are sparse in the literature.

As already mentioned, the problem with constipation among old people is significant and is probably often overlooked. An earlier study conducted in the same population as the present study found that the use of opioids increased between 2007 and 2013 [17]. Considering the increased risk of constipation with the use of opioids, an increased prevalence of constipation and use of laxatives could be expected between the years. Thus, the aim of this study was to compare the prevalence of constipation and use of laxatives between 2007 and 2013 and to analyze constipation and laxative use among people who were prescribed opioids. The aim was also to identify any factors associated with constipation.

## Methods

### Material

The data for this project were collected from a questionnaire distributed to all those living in nursing homes in the county of Västerbotten in Northern Sweden. The questionnaires were distributed in 2007 and 2013 with response rates of 85.8 and 70.5%, respectively. In 2007, information about 3070 persons was collected and in 2013 information about 2262 persons was collected. Geriatric and psychogeriatric hospital wards were included in the survey in 2007, but not in 2013. Thus, 99 persons for 2007 were excluded. In addition, people under 65 years of age or for whom no age was registered were excluded from the analysis for both years, leaving 2820 people from 2007 and 2135 people from 2013. Finally, those for whom no information about the use of laxatives was registered were excluded. Thus, 2820 people from 2007 and 1902 people from 2013 were selected for the analysis.

### Procedures

The questionnaire used, the Multi-Dimensional Dementia Assessment Scale (MDDAS), included instructions on how to make the assessments based on observations over one week. The staff who knew the resident best were asked to complete that resident’s form [18]. The MDDAS has good inter- and intra-rater reliability [18]. The MDDAS includes assessments of cognition and activities of daily living (ADL) and questions regarding, for example, pain and constipation. Registration of current drug prescriptions is also included.

An ADL score was calculated based on the resident’s ability to cope with hygiene, dressing, eating and bladder and bowel control [19]. All ADL items were scored 1–5, apart from bladder control, which was scored 1–4. The ADL score has a range of 4–24, where a higher number represents greater ADL independence.

Cognitive impairment was measured using a scale developed by Gottfries and Gottfries [19, 20] comprising 27 items that measure a person’s level of cognitive function. A score of less than 24 points indicates cognitive impairment, correlating with a sensitivity of 90% and specificity of 91% [18] to the usual 24/30 Mini-Mental State Examination (MMSE) [21] cut-off.

#### Definition of constipation

Assessments regarding constipation were based on observations over one week. The following questions were included in the MDDAS; (i) “Does the resident have hard stools?”, (ii) “Is it strenuous for the resident to defecate?”, and (iii) “Does the resident defecate less than three times per week?”. The resident was rated as being constipated if one or more of these questions were answered with a “yes”.

#### Information regarding drugs

Information regarding drug use was collected as part of the MDDAS and the drug data were then grouped and coded by members of the research team. Current drug prescriptions at the time of collection were registered. The WHO ATC (Anatomical Therapeutic Chemical Index) classification system was used to group the drugs. The following drugs and drug classes were included in this analysis: A06A (laxatives) with the subgroups A06AB (stimulant laxatives), A06AC (bulking agents), A06AD (osmotic laxatives), A06AG (rectally-administered laxatives – bisacodyl, sorbitol, docusate and sodium citrate) and A06AX (other drugs). Further, N02A (opioids) and anticholinergic drugs, classified according to the Swedish National Board of Health and Welfare [22], were included in the analysis. The definition includes drugs from different ATC classes, for example, some antipsychotic and antidepressant drugs. Information regarding doses was not coded. Further, information regarding pro re nata drugs (“as needed”) was not coded. Hence, only regular use of the drugs was analyzed.

### Statistics

Basic characteristics among the residents in 2007 and 2013 were compared using the Pearson chi-square test and the independent sample *t*-test. To compare different factors between the years in regression models, constipation symptoms and laxative, opioid and anticholinergic drug use respectively were dependent variables in each model. Year (2007 or 2013), sex, age, level of ADL dependency and cognitive function were all independent variables in the different models. To identify factors associated with constipation, a multiple logistic regression model was constructed. The model had constipation as the dependent variable and included sex, age, level of ADL dependency, cognitive function, year of investigation (2007 or 2013), opioids and anticholinergic drugs as independent variables. Finally, people prescribed opioids were selected and the prevalence of constipation symptoms and laxative use was compared between the two respective years using the Pearson chi-square test. All statistical calculations were performed using SPSS v25 and a *p*-value < 0.05 was considered statistically significant.

## Results

Table 1 shows the characteristics of the study population in 2007 and 2013. There was no difference in the basic characteristics except for age, where people were significantly older in 2013 compared to 2007.

**Table 1:** Basic characteristics of the study population

	2007	2013	*p*-value
Total number of residents *n*	2820	1902	
Women *n* (%)	1924/2814 (68.4)	1291/1887 (68.4)	0.975
Age, mean ± SD	84.6 ± 6.8	85.1 ± 7.0	0.027
Residents with cognitive impairment *n* (%)	1971/2770 (71.2)	1362/1861 (73.2)	0.131
ADL score (4–24), mean ± SD	15.5 ± 6.3	15.6 ± 6.2	0.611

*ADL* activities of daily living, *SD* standard deviation

In 2007, 36% of the residents were constipated according to the definition used compared to 40% in 2013. After correcting for sex, age, ADL performance and level of cognitive impairment, this difference was found to be statistically significant (*p* = 0.032). There were no significant differences in the number of affirmative answers to the questions “Does the resident have hard stools?” and “Is it strenuous for the resident to defecate?” between 2007 and 2013, while answering yes to “Does the resident defecate less than three times per week” was significantly more common in 2013 (Table 2).

**Table 2:** Constipation symptoms among residents in 2007 and 2013

	2007	2013	Odds ratio^a^	95% confidence interval^a^	*p*-value^a^
*n*	2820	1902			
The resident is constipated^b^ n (%)	994/2744 (36.2)	697/1763 (39.5)	1.170	1.013–1.350	0.032
The resident has hard stools *n* (%)	542/2745 (19.7)	383/1783 (21.5)	1.146	0.972–1.350	0.104
It is strenuous to defecate *n* (%)	562/2753 (20.4)	360/1783 (20.2)	0.985	0.833–1.165	0.860
The resident defecates less than three times per week *n* (%)	503/2751 (18.3)	388/1778 (21.8)	1.334	1.128–1.577	0.001

^a^Corrected for sex, age, activities in daily living performance and level of cognitive impairment. ^b^Defined as constipated if one or more of the three questions; (i) “Does the resident have hard stools?”, (ii) “Is it strenuous for the resident to defecate?”, and (iii) “Does the resident defecate less than three times per week?” were answered with a “yes”

The overall use of laxatives increased between 2007 (46%) and 2013 (59%). The difference was statistically significant after correction for sex, age, ADL performance and level of cognitive impairment (Table 3). Within the drug class, stimulant laxatives increased significantly from 10 to 14% and osmotic laxatives from 41 to 53%. Further, opioid use increased and the use of anticholinergic drugs decreased between 2007 and 2013.

**Table 3:** Laxative, opioid and anticholinergic drug use in 2007 and 2013

	2007	2013	Odds ratio^a^	95% confidence interval^a^	*p*-value^a^
*n*	2820	1902			
Laxatives (A06A), *n* (%)^b^	1297 (46.0)	1129 (59.4)	1.728	1.512–1.976	< 0.001
Stimulant laxatives (A06AB) *n* (%)	289 (10.2)	265 (13.9)	1.446	1.182–1.768	< 0.001
Bulking agents (A06AC) *n* (%)	60 (2.1)	46 (2.4)	1.086	0.706–1.670	0.706
Osmotic laxatives (A06AD) *n* (%)	1167 (41.4)	1002 (52.7)	1.584	1.388–1.807	< 0.001
Rectally-administered laxatives (A06AG) *n* (%)	77 (2.7)	43 (2.3)	0.811	0.541–1.215	0.310
Opioids (N02A) *n* (%)	551 (19.5)	485 (25.5)	1.365	1.169–1.592	< 0.001
Anticholinergic drugs^c^ *n* (%)	381 (13.5)	170 (8.9)	0.639	0.517–0.788	< 0.001

^a^Corrected for sex, age, activities in daily living performance and level of cognitive impairment. ^b^“Other laxative drugs” are included in A06A but not reported separately because of low prevalence. ^c^According to the Swedish National Board of Welfare

In the multiple logistic regression model, there was an increased risk of having constipation in 2013 compared to 2007. Furthermore, people being prescribed opioids and anticholinergic drugs, respectively were at increased risk of being constipated. In contrast, a higher ADL score was associated with a decreased risk of constipation (Table 4).

**Table 4:** Logistic regression analysis of factors associated with constipation^a^

	Simple OR (95% CI)	Multiple OR (95% CI)
Year 2013^b^	1.151 (1.018–1.302)	1.162 (1.006–1.342)
Older age	1.002 (0.994–1.011)	
Female sex	1.123 (0.985–1.279)	
Higher ADL score	0.885 (0.875–0.895)	0.883 (0.870–0.896)
Higher cognitive score^c^	0.949 (0.941–0.956)	1.002 (0.991–1.012)
People prescribed opioids	1.748 (1.516–2.017)	1.610 (1.361–1.905)
People prescribed anticholinergic drugs	1.440 (1.200–1.727)	1.264 (1.020–1.564)

^a^Defined as constipated if one or more of the three questions; (i) “Does the resident have hard stools?”, (ii) “Is it strenuous for the resident to defecate?”, and (iii) “Does the resident defecate less than three times per week?” were answered with a “yes”. ^b^Reference year 2007. ^c^Cognitive score ranges from 0 to 27 points and a score of less than 24 is considered to indicate cognitive impairment. The multiple model includes significant variables from the simple model. ADL, activities in daily living; CI, confidence interval; OR, odds ratio

In 2007, 551 people (20%) were prescribed opioids and the corresponding number for 2013 was 485 (26%) (Table 3). Among these, there was a statistically significant difference in the use of laxatives between the years, 57% in 2007 compared to 77% in 2013. There was also a statistically significant difference in the number of persons rated as being constipated and not using laxatives between 2007 (35%) compared to 2013 (20%) (Table 5).

**Table 5:** Constipation and laxatives among people with prescribed opioids in 2007 and 2013

	2007	2013	Total	*p*-value
*n*	551	485	1036	
The resident is constipated^a^ *n* (%)	248/535 (46.4)	226/452 (50.0)	474/987 (48.0)	0.253
The resident has hard stools *n* (%)	136/531 (25.6)	139/458 (30.3)	275/989 (27.8)	0.097
It is strenuous to defecate *n* (%)	171/536 (31.9)	137/458 (29.9)	308/994 (31.0)	0.499
The resident defecates less than three times per week *n* (%)	120/538 (22.3)	133/455 (29.2)	253/993 (25.5)	0.013
Laxatives (A06A), *n* (%)^b^	316 (57.4)	374 (77.1)	690 (66.6)	< 0.001
Stimulant laxatives (A06AB) n (%)	107 (19.4)	124 (25.6)	231 (22.3)	0.018
Bulking agents (A06AC) *n* (%)	15 (2.7)	8 (1.6)	23 (2.2)	0.242
Osmotic laxatives (A06AD) *n* (%)	280 (50.8)	325 (67.0)	605 (58.4)	< 0.001
Rectally-administered laxatives (A06AG) *n* (%)	20 (3.6)	13 (2.7)	33 (3.2)	0.385
Residents rated as constipated without laxatives, n (%)	86/248 (34.7)	45/226 (19.9)	131/474 (27.6)	< 0.001

^a^Defined as constipated if one or more of the three questions; (i) “Does the resident have hard stools?”, (ii) “Is it strenuous for the resident to defecate?”, and (iii) “Does the resident defecate less than three times per week?” were answered with a “yes”. ^b^“Other laxative drugs” are not reported separately because the prevalence in this group was zero

## Discussion

This study found that the prevalence of constipation and the use of laxatives among people living in nursing homes increased between 2007 and 2013. People prescribed opioids and anticholinergic drugs were at increased risk of constipation, while people with a higher ADL score were at decreased risk. Further, there was a decrease between the respective years in the number of people not prescribed laxatives for regular use among those prescribed opioids and rated as constipated.

Due to differences in definition, the prevalence of constipation in 2007 in the present study (36%) was lower than the prevalence found among the same study population in a previous investigation (67%) [23]. In general, the prevalence of constipation is difficult to compare between different studies because several definitions are used. Laxative use has often been used as a marker for constipation, either independently or in combination with other criteria [3, 24]. However, because one of the aims of the present study was to analyze the use of these drugs, we chose not to include laxatives in the definition, and this is the reason why the prevalence is lower in the present study compared to the study mentioned above [23]. In clinical practice, constipation is often defined as fewer than three bowel movements per week [25] and this symptom was also the only single symptom that increased significantly between the years in the present study. This symptom is also included in the Rome IV Criteria [26]. Two of the other six symptoms in these criteria are “straining during more than 25% of defecations” and “lumpy or hard stools in more than 25% of defecations”. The presence of two or more symptoms for the last three months and some additional criteria are required to fulfil the criteria for constipation according to this definition. Other organizations, such as the American Gastroenterological Association, recommend broader definitions of chronic constipation [5]. In the present study, we defined constipation as having at least one of the three symptoms: (i) “Does the resident have hard stools?”, (ii) “Is it strenuous for the resident to defecate?”, and (iii) “Does the resident defecate less than three times per week?”

In the present study, the regular use of laxatives increased between 2007 and 2013, but despite this, the prevalence of constipation increased between the two respective years. This increase in constipation was independent of opioid use or anticholinergic use. Nevertheless, being constipated was associated with both of these drug classes in the present study. Anticholinergic drugs decreased between 2007 and 2013 which, in turn, could be a consequence of current guidelines [22]. Anticholinergic drugs are not recommended for older people because of adverse drugs effects which, in addition to constipation, include dry mouth, urinary retention, confusion and memory impairment [27].

The other drug class associated with constipation was opioids. The experience of pain is highly prevalent among nursing home residents [17] and the increase in opioid use probably reflects current recommendations in which opioids (with the exception of some weak opioids such as tramadol) are recommended for the treatment of pain in fragile elderly people before the use of, for example, nonsteroidal anti-inflammatory drugs [22, 28]. Considering the risk of constipation among people prescribed opioids, it is positive that the number of people taking laxatives has increased in this group. Nevertheless, among people prescribed opioids and rated as constipated, 35% in 2007 and 20% in 2013 were not prescribed laxatives for regular use. Prevention should be the preferred strategy [22] and this could be of particular importance among people with dementia who might have difficulties in recognizing and communicating adverse drug events like constipation. In the present study, constipation was associated with a lower cognitive and ADL score in the simple regression model and with a lower ADL score in the multiple regression model. Even if problems with recognizing and communicating adverse drug events might be more relevant among people living at home without help, this issue is important to pay attention to in a nursing home context because these kinds of problems could even lead to hospitalization among people with dementia [29].

Another reason for increased constipation could be the choice of laxatives. However, the choice of laxatives among the residents in this study appears to be rational for both years. The use of bulking agents was very low in both years, also among people who were prescribed opioids. These drugs are unsuitable for OIC because opioids prevent peristalsis of the fiber-increased bulk, which could in some cases contribute to bowel obstruction [11]. Further, the use of osmotic laxatives and stimulant laxatives increased significantly between the years, which is also positive. Studies have demonstrated the positive effects of osmotic laxatives such as PEG in treating chronic constipation, and adverse effects have been judged to be minimal [30]. Also, stimulant laxatives given regularly do not appear to lead to structural or functional impairment of the colon, contrary to former beliefs [31, 32]. The fact that the use of osmotic laxatives was most common in the present study is consistent with two previous studies conducted in the Nordic countries among people living in nursing homes [3, 15], while one study from the UK found that stimulant laxatives were the most commonly used laxatives [13].

The strengths of the present study include the large, unselected sample of people living in nursing homes in 2007 and 2013. Drug registration was of a high quality. However, the lack of data on pro re nata prescription could lead to an underestimation of the actual use of laxatives because these drugs are sometimes used intermittently rather than daily.

The constipation assessment used in this study was not formulated completely according to the Rome Criteria or other recommended definitions, which would have been desirable in order to be able to compare the results with other studies. However, the questions used formed part of the Rome Criteria. Some of the other questions in the Rome Criteria would have been difficult to use because part of the criteria is based on perceived sense, which could be hard to communicate among this population that has a high prevalence of cognitive impairment. The fact that many of the residents suffered from cognitive impairment is also the reason why the questionnaire was completed not by the residents themselves but by a staff member. Nevertheless, even though the questionnaire was answered by a staff member who knew the resident best (i.e. the resident’s key worker), it is still a proxy estimate and a possible source of error compared to direct measurements. In addition, the observation period was only one week and therefore only reflects the symptoms the person exhibited during this period. Further, according to the definition of constipation used in this study, residents who were satisfactorily treated for constipation have not been classified as having constipation. This should be taken in account when interpreting the results. However, because the same criteria were used in 2007 and 2013 in this study, the comparison between the years can be considered relevant.

## Conclusion

This study found that the prevalence of symptoms of constipation increased between 2007 and 2013. The use of opioid drugs and anti-cholinergic drugs was associated with an increased risk of constipation. The use of laxatives increased between the years. However, there were still a number of people being prescribed with opioids and rated as constipated who were not treated with laxatives. This study therefore indicates that constipation remains a significant problem among people in nursing homes and also indicates that those prescribed opioids could benefit from an increased awareness of the risk of constipation and treatment, if required.

## Abbreviations

ADL: Activities of Daily Living
MDDAS: Multi-Dimensional Dementia Assessment Scale
MMSE: Mini-Mental State Examination
OIC: Opioid-induced constipation
PEG: Polyethylene glycol
SD: Standard deviation
SPSS: Statistical Package for Social Science
